# Can the second law of thermodynamics hold in cell cultures?

**DOI:** 10.3389/fgene.2015.00262

**Published:** 2015-08-07

**Authors:** Kumar Selvarajoo

**Affiliations:** ^1^Institute for Advanced Biosciences, Keio UniversityTsuruoka, Japan; ^2^Systems Biology Program, Graduate School of Media and Governance, Keio UniversityFujisawa, Japan

**Keywords:** self-organization, entropy, cell dynamics, thermodynamics, computational biology

Living systems have the ability to adapt and self-organize when challenged with drastic environmental changes. The remarkable characteristic of plasticity and collectivity allow them to evolve and survive over billions of years in a rather unpredictable manner. Observing and studying the dynamic complexity have influenced many scientists across diverse disciplines to believe that living systems operate far from equilibrium and, hence, the second law of thermodynamics and ergodicity breaks down (Stuart, 1995; Prigogine, 1997). Therefore, it may not be feasible to develop simple deterministic models to interpret complex living systems' behavior.

Briefly, the second law of thermodynamics states that entropy in systems that are in equilibrium will increase over time or space. In other words, order will decrease in a thermodynamically equilibrium system where there is no exchange of matter or energy. Living systems, which constantly exchange matter and energy to the surroundings, can be considered to exist far from equilibrium to achieve biological order (Stuart, 1995). One appropriate example is the ability of bacteria to exchange pheromone during environmental threats, such as during antibiotic treatment, to form biofilms which are highly organized structures resistant to the therapeutic intervention (Chatterjee et al., 2013). The biofilm example demonstrates that the cooperative behavior of organisms can be very different to the individual response. Thus, using ergodic principle or predictive deterministic approaches to understand cellular behaviors can be questionable, and this issue has been debated from time to time.

Most biological experimentations of mammalian cells are performed *in vitro*, where cells from living tissues are removed from their physiological neighbors and regrown in minimum media that will support the morphology, survival, and growth of the cells. The number of cells used in different experiments, although variable, are usually several order of magnitudes lower than that in actual tissues or organs. Under such far from realistic laboratory conditions, are *in vitro* cells able to display emergent behaviors?

Remarkably, genome-wide oscillatory behaviors have been observed in continuously cultured laboratory yeast, and the mammalian circadian clocks have been reproduced in a plate (Klevecz et al., 2004; Sato et al., 2006). Although fascinating, the collective behaviors were achieved for limited periods under carefully controlled experimental conditions, such as the rate of aeration and agitation of fermenters, etc. Outside the specified range, the synchronization of cells fade. In a more recent effort, cultured, and self-assembling engineered human cardiac tissue created rhythmic heart beat that was highly similar to the human heart (Turnbull et al., 2014). Therefore, from these examples, it becomes conceivable that during *in vitro* laboratory experimentations, complex non-linear and self-organizing behaviors can still be achieved if the technical and environmental conditions are carefully and tightly managed to mimic the actual reality, considering the exchange of key matter between the surroundings.

Today, in the name of systems biology approaches, we have seen numerous works that have employed theoretical models to interpret and predict cellular responses. It is surprising to note that despite the complexity of living systems, numerous deterministic models have been rather successfully used to understand both linear and non-linear responses (Selvarajoo, 2014). A vast majority of cellular models are based on ordinary differential equations, mass-action kinetics, Michaelis–Menten kinetics or Boolean logics. In most circumstances, if not all, the investigations considered “closed” system modular approach, where the models did not include continuous exchange of materials between the internal and external environments and, hence, chemical and thermal equilibrium have been assumed. That is, the approaches often adopted well-mixed, homogenous and isothermal environment where each reaction in the cellular network is connected through first-order, higher-order mass-action, enzyme kinetic equations, or simply Boolean logics, depending on the knowledge gained for individual reaction.

Despite the simplicity, these models, combined with experimental verifications, have progressed our understanding of several complex mechanisms controlling cell processes. For example, the elucidation of distinct feedback mechanisms in epidermal- and nerve- growth factor signaling utilizing the same MAP kinases module (Santos et al., 2007), and the identification of key target for enhancement of apoptosis in cancers (Piras et al., 2011; Hayashi et al., 2015). In several other studies, even without the need to know graded response, simple binary (ON/OFF) state approaches utilizing discrete Boolean network modeling have produced insightful results in understanding diseases processes (Benenson et al., 2004; Zhang et al., 2008; Schlatter et al., 2009). More recently, even immune cell divisions based on main and co-stimuli have induced responses that can be shown to be linear functions of their signaling components (Hawkins et al., 2013; Marchingo et al., 2014). So, if deterministic and rule-based models can be applied successfully using *in vitro* dataset, does it indicate that ergodic hypothesis could hold in 2-D plated cell cultures? That is, under the conditions where simple models work, are cells experiencing thermodynamic equilibrium?

Here, it is noteworthy to give some examples from our research on analyzing *in vitro* large scale time-series gene expressions dataset of mammalian innate and adaptive immune cell types to distinct environmental stimulations (Tsuchiya et al., 2009; Selvarajoo and Giuliani, 2012; Simeoni et al., 2015). Using simple statistical approaches of Pearson/Spearman correlations, Shannon entropy, Mutual Information and noise (squared coefficient of variation), in summary, we found that these metrics changed from an initial value at stimulation to achieve stable values at later times. More precisely, we noticed the levels of transcriptome-wide expression disorder increased after appropriate immune stimulation, and eventually reached asymptotic values. (This can be analogous to a tiny drop of ink added to water will show increasing disorder in color, and eventually stabilizes). In another recent study (Piras et al., 2014), on tracking transcriptome-wide variability in mammalian developmental cells, we also observed the increase in gene expressions disorder across the developmental process, reaching stable values at later stages (Figure 1). The observations from these *in vitro* studies indicate that cultured cells with fixed stimulation and condition may be reaching a state that is indicative of pseudo equilibrium.

**Figure 1:**
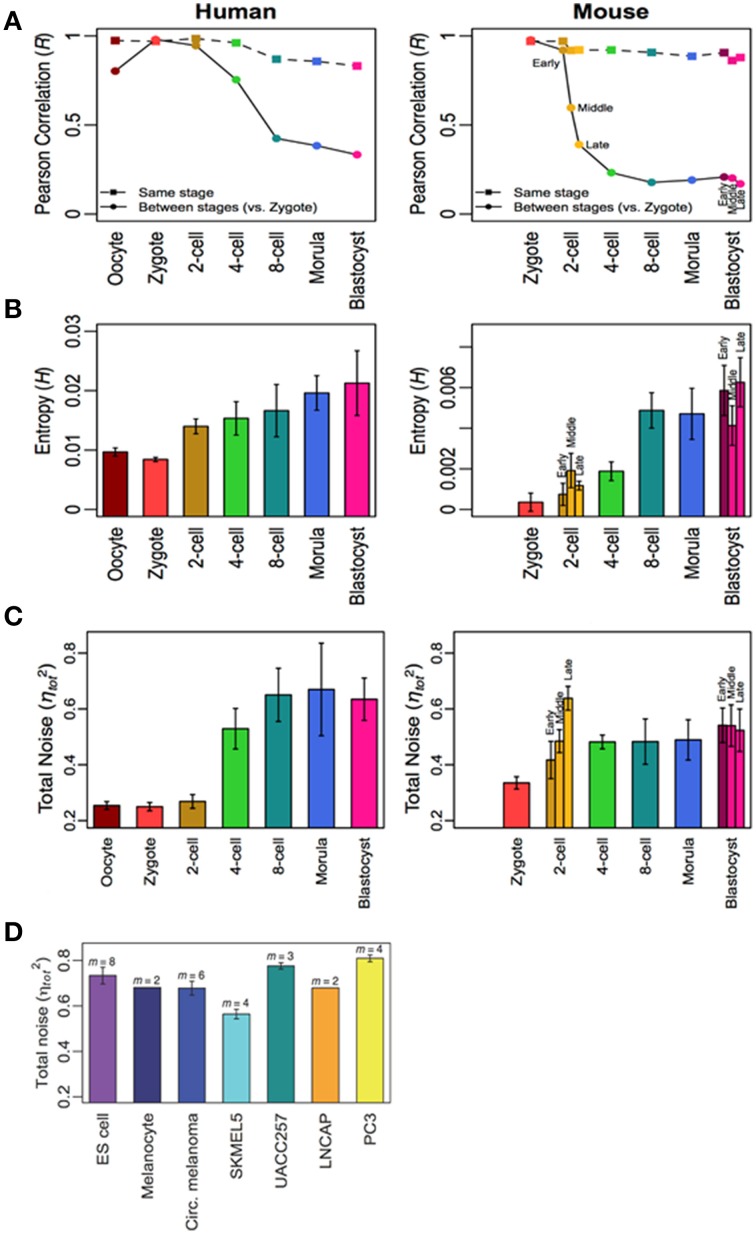
**Increasing transcriptome-wide disorder for human and mouse development cells. (A)** Pearson correlation, **(B)** Shannon entropy, and **(C)** noise from oocyte to blastocysts indicate increasing disorder which stabilizes, and is similar to other differentiated and cancer cells **(D)**. Figure adapted from Piras et al. (2014).

On a different note, we monitored the transcriptome-wide expression variability from single cells to cell population in increasing ensemble sizes across six mammalian cell types (Piras and Selvarajoo, 2015). We observed that while increasing cell ensemble size, transcriptome-wide noise reduced approximately following the law of large numbers. Furthermore, the entire gene expressions of cell populations (and only the highly expressed portion of single cells) followed the central limit theorem. More simply, unlike single cells, which are probably more vulnerable to stochastic noise (Elowitz et al., 2002; Selvarajoo, 2012), cell populations showed reduced transcriptome-wide noise. It is, therefore, conceivable that deterministic rules can be utilized and modeled for the stable population-wide average response. However, for single cell responses stochastic modeling have been shown to be most appropriate, consider the investigations on bistable cell fates in bacteria (Dubnau and Losick, 2006). Nevertheless, both single cell stochastic and population-wide deterministic modeling based on *in vitro* data exclude or disregard the exchange of matter between other diverse cell types or the natural environment. This is especially important for multicellular organisms, where information is constantly exchanged within the entire system.

To summarize, it has been debated for a long time whether living systems can be mathematically conceptualized using simple theories as they possess very complex dynamic and emergent behaviors, and many times display unpredictable outcomes. Interestingly, certain self-organizing and non-ergodic behaviors have been observed even in laboratory conditions. In the case of an oscillating system, we can set up experiments and define conditions to mimic *in vivo*-like response of molecular dynamics. Whereas, in other situations, it may be very difficult to define experimental conditions that consider far from thermodynamic equilibrium phenomenon observed in self-organizing *in vivo* systems. Under such conditions, simple statistical techniques on large-scale time series omics dataset can be used to monitor multi-dimensional entropy and variability that could provide hints into the global state of the system (Piras et al., 2014). Notably, we have highlighted that tracking the global responses of living cells, *in vitro*, have shown a general increase in entropy and variability from initial values, which stabilize at higher levels at later time points. Under such stabilizing entropy condition, it is our opinion that deterministic models can continue to play vital roles for *in vitro* studies. Nevertheless, for *in vivo* systems, the rules for self-organization require further investigations (Stuart, 1995), especially for multi-cellular organisms considering the exchange of matter between different cell types. Knowing this will allow us to better predict the emergent properties of the “real” living systems.

## Conflict of interest statement

The author declares that the research was conducted in the absence of any commercial or financial relationships that could be construed as a potential conflict of interest.
